# The Role of the MCTS1 and DENR Proteins in Regulating the Mechanisms Associated with Malignant Cell Transformation

**DOI:** 10.32607/actanaturae.11181

**Published:** 2021

**Authors:** E. Y. Shyrokova, V. S. Prassolov, P. V. Spirin

**Affiliations:** Engelhardt Institute of Molecular Biology, Russian Academy of Science, Moscow, 119991 Russia; Moscow Institute of Physics and Technology (National Research University), Dolgoprudny, Moscow Region, 141701 Russia

**Keywords:** MCTS1 and DENR proteins, malignant cell transformation, translation initiation factors, signaling pathways, apoptosis, cell cycle

## Abstract

The mutations associated with malignant cell transformation are believed to disrupt the expression of a significant number of normal, non-mutant genes. The proteins encoded by these genes are involved in the regulation of many signaling pathways that are responsible for differentiation and proliferation, as well as sensitivity to apoptotic signals, growth factors, and cytokines. Abnormalities in the balance of signaling pathways can lead to the transformation of a normal cell, which results in tumor formation. Detection of the target genes and the proteins they encode and that are involved in the malignant transformation is one of the major evolutions in anti-cancer biomedicine. Currently, there is an accumulation of data that shed light on the role of the MCTS1 and DENR proteins in oncogenesis.

## INTRODUCTION

It is a generally accepted fact that mutations associated with malignant cell transformation disrupt the expression of a significant number of genes whose protein products are involved in the regulation of the activity of many signaling cascades. These cascades are associated with the mechanisms responsible for differentiation, proliferation, as well as sensitivity to apoptotic signals, growth factors, and cytokines.

Abnormalities in the balance of signaling cascades can lead to cell transformation, and subsequent tumor formation. The search for the target genes – and their encoded proteins – which are involved in malignant cell transformation is one of the main challenges of modern cancer biomedicine. Currently, a growing body of data indicates that these genes include *MCTS1 *and* DENR*.

## MCTS1 AND DENR EXPRESSION

The *MCTS1* (Malignant T-cell-amplified sequence 1) gene, located on the long arm of the X chromosome (Xq22-24), was first described in 1998, at the same time the hypothesis about its involvement in the development of malignant diseases, in particular, the malignant transformation of T-cells, was proposed [1]. Later, the MCTS1 protein was shown to possess the RNA-binding domain PUA, which is characteristic of some tRNA- and rRNA-binding proteins [2]. Next, the PUA domain of MCTS1 was found to be involved in the interaction with the cap-binding complex, one of the components of which, namely the DENR protein, contains the SUI1 domain, which is responsible for translation initiation [3, 4, 5].

It is now known that both proteins are normally expressed in almost all tissues; however, the mechanisms they regulate have not been established yet. MCTS1 is assumed to be involved in the regulation of various processes, including cell cycle modulation and apoptosis induction. The gene coding for the DENR (Densityregulated re-initiation and release factor) protein is located on the long arm of chromosome 12 (12q24.31). DENR got its name after a close correlation was uncovered between its level and cell density in culture [6]. The 3’-untranslated region (3’-UTR) of DENR mRNA contains adenine- and uracil-rich sequences. These sequences often serve as binding regions for some of the proteins involved in mRNA turnover. In particular, the AUF1 ribonucleoprotein can bind adenine/uracil-rich regions of the DENR mRNA 3’-UTR, and inhibition of AUF1 expression by RNA interference increases the DENR protein level in cells [7, 8, 9].

It has been established relatively recently during ribosomal profiling of NIH3T3 cells with *DENR *knockdown that this protein can bind to the upstream open reading frame (uORF) of *CLOCK *mRNA, one of the key regulators of circadian rhythms [10, 11]. This led to the conclusion that DENR may also be one of the proteins potentially involved in regulating cyclic fluctuations in the biological processes associated with alteration of day and night. Laboratory mice studies showed that the DENR and MCTS1 proteins are involved in neuronal migration during brain development. Furthermore, the *DENR *mutations p.C37Y and p.P121L, resulting in abnormal protein forms, are found in the neuronal cells of patients with autism and Asperger’s syndrome, respectively [12].

## MCTS1 AND DENR IN TRANSLATION REGULATION

As mentioned above, the involvement of the MCTS1 and DENR proteins in translation regulation has been studied the most. Recently, it has been shown that the MCTS1–DENR complex is highly homologous to the translation initiation factor eIF2D [13]. The MCTS1– DENR complex plays an important role in translation re-initiation [14, 15, 16, 17, 18]. Eukaryotic translation re-initiation can occur when the ribosome initiates translation at the uORF. This results in translation termination, with its subsequent re-initiation at the main ORF [19]. However, the molecular mechanisms regulating translation re-initiation are still poorly understood. There are several factors known to be involved in re-initiation; they include the canonical translation initiation factors eIF1, eIF2, and eIF3, which remain associated with ribosomes after termination at uORFs [20]. Later, it was found that eIF2D, a larger protein with a MCTS1 and a DENR homology domains in the N-terminal and C-terminal regions, respectively, is involved in translation re-initiation [14, 15].

**Figure 1:**
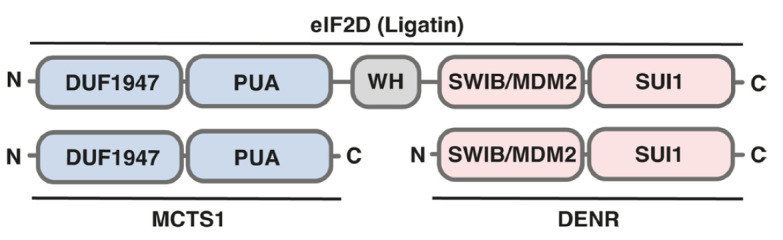
Domain structure of DENR, MCTS1, and eIF2D. DUF1947 – domain with unknown function; PUA – RNA-binding domain; SWIB/MDM2 – regions homologous to the SWIB protein involved in chromatin remodeling and the p53 inhibitor MDM2; SUI1 – protein region functionally similar to the initiation factor eIF1; WH (winged helix) – DNA-binding domain. MCTS1-homologous regions are highlighted in blue. DENR-homologous regions are highlighted in pink

*Figure 1* shows a schematic representation of the homologous domains between the proteins.

The involvement of DENR and MCTS1 in translation re-initiation was demonstrated in various models, including human cells [18, 21]. Translation re-initiation is known to be accompanied by the formation of a heterodimeric MCTS1–DENR complex and its binding to tRNA [22]. During translation re-initiation, the MCTS1–DENR heterodimer binds to the small (40S) ribosomal subunit, with direct interaction between MCTS1 and the h24 helix of 18S rRNA and between the DENR C-terminal region and the h44 helix of 18S rRNA. This interaction is believed to result in tRNA recruitment to the P-site of the 40S ribosomal subunit. X-ray crystallography studies of the C-terminal region in DENR revealed a high degree of homology between this protein and initiation factor eIF1 [23], which also indicates the involvement of DENR in translation regulation.

## MCTS1 IN THE REGULATION OF CELL CYCLE AND CDK4/6 ACTIVITY

**Fig. 2:**
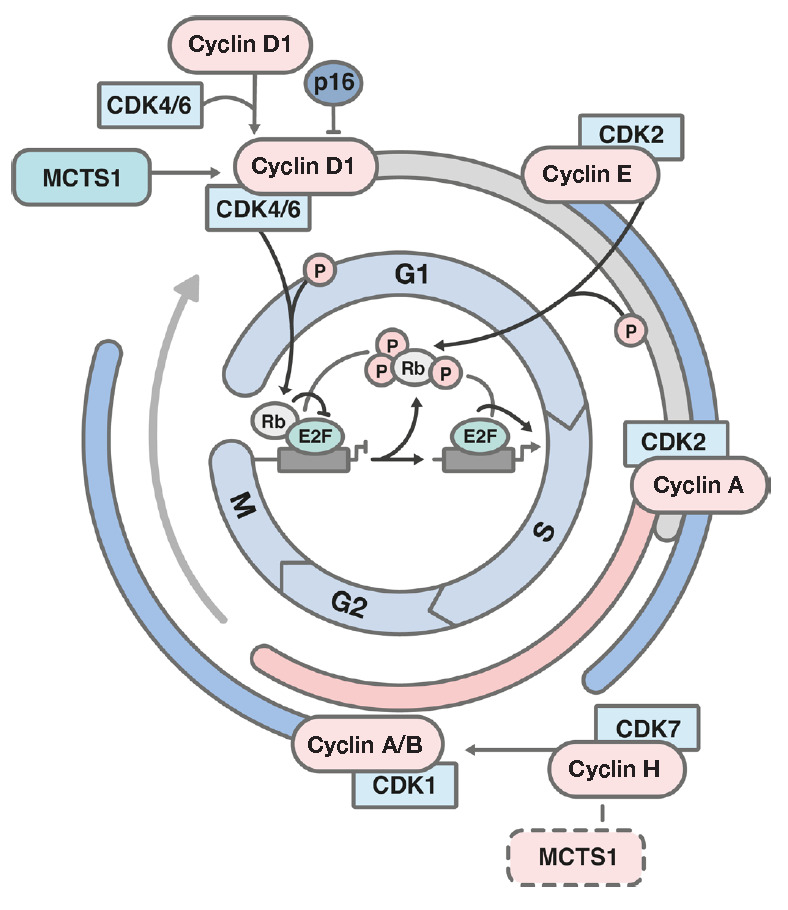
Schematic representation of the cell cycle. CDK – cyclin-dependent kinase; it is involved in cell cycle progression. Phosphorylation of the Rb (retinoblastoma protein) protein leads to transition through the G1/S stages. E2F – transcription factor; p16 (CDKN2A) – a CDK inhibitor; it impedes cell division while inhibiting G1/S transition; G1/S/G2 – interphase, M – mitosis

The MCTS1 protein is involved in cell cycle regulation.* MCTS1 *overexpression was shown to increase the proliferation rate of NIH3T3 cells; in particular, by accelerating the progression of the G1 phase of the cell cycle. Meanwhile, this stimulates cell growth [1]. Analysis of cell growth in a semi-liquid medium showed that only cells overexpressing *MCTS1 *can form viable colonies [1, 24, 25]. Ectopic expression of *MCTS1 *in interleukin-2- (IL-2-)-dependent human EC155 T-cells sensitize them to apoptotic signals [24]. G1 phase progression involves type D- and E-type cyclins, as well as cyclin-dependent kinases (CDKs). Cyclins-D forms a complex with either CDK4 or CDK6 (*Fig. 2*) [26, 27, 28, 29, 30]. Ectopic expression of* MCTS1 *in NIH3T3 cells increases the level of cyclin D and the efficiency of cyclin D/CDK4 and cyclin D/CDK6 complex formation [24].

A region with a degree of homology to the sequence encoding for cyclin H, namely, a domain responsible for protein-protein interactions, was found in the *MCTS1* nucleotide sequence [31]. This homology between MCTS1 and cyclin H may indirectly indicate the involvement of the MCTS1 protein in cell cycle regulation, particularly the mitotic phase.

## MCTS1 AND REGULATION OF APOPTOSIS

MCTS1 is known to reduce the intracellular level of the p53 and p21 proteins, which can also contribute to malignant cell transformation and promote tumorigenesis [31]. Treatment of human MCF-7 cells with bleomycin, which induces double-strand breaks in the DNA of rapidly dividing cells, increases the expression of *TP53* encoding the p53 protein. Ectopic *MCTS1 *expression decreases the level of p53 activation in cells treated with bleomycin and, hence, the efficiency of apoptosis of damaged cells [31].

Cells with ectopic expression of *MCTS1 *contain higher levels of ubiquitinated p53 (Ub–p53) and phosphorylated MDM2. This suggests that a decrease in the p53 level due to high *MCTS1 *expression may be associated with MDM2-dependent degradation of p53 in proteasomes [32]. Treatment of these cells with the proteasome inhibitor MG132 increased the p53 level, which indicates the involvement of MCTS1 in the regulation of its stability [31].

**Fig. 3:**
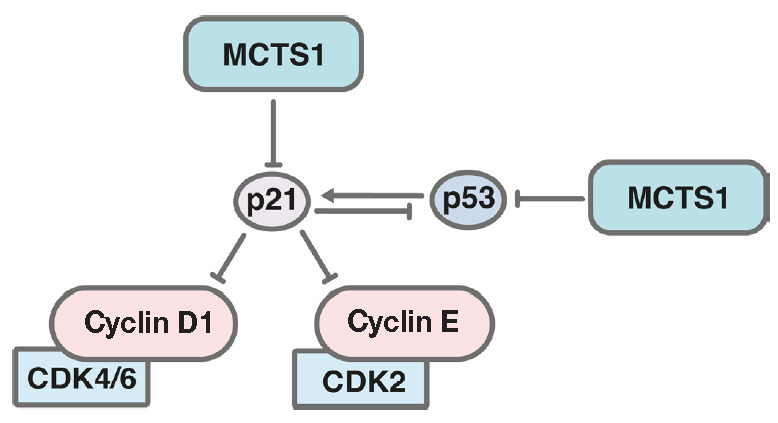
Effect of MCTS1 on the pro-apoptotic protein p53 and its inhibitor p21. Formation of a complex between cyclin D1 and CDK4/6 and a complex between cyclin E and CDK2 regulates the transition through the G1 stage of the cell cycle

Treatment of cells with ectopic expression of *MCTS1* with bleomycin resulted in a less efficient synthesis of the p21 protein, one of the major targets of p53, compared to control cells. Small interfering RNA-mediated suppression of *MCTS1 *increased the expression levels of not only p53, but also p21 (*Fig. 3*) [31]. The MEK/ ERK signaling cascade is known to be involved in the regulation of p53 activity and p21 expression [33, 34]. MCTS1 enhances phosphorylation of the ERK1/2 protein kinase (pMAPK) [35], which is part of one of the main signaling pathways involved in malignant cell transformation and associated with sensitivity to chemotherapeutic drugs [36, 37, 38, 39]. Inhibition of *MCTS1* expression by RNA interference in MCF-10A breast cancer cells, and A549 lung cancer cells, results in caspase- 3 activation and cell death. Suppression of *MCTS1* expression in lung and breast tumors xenografts significantly suppresses tumor development [35, 40].

## ASSOCIATION OF MCTS1 WITH CHROMOSOME INSTABILITY

Cytogenetic analysis demonstrated that MCTS1 affects genome integrity. In particular, irradiated MCF-7 cells overexpressing *MCTS1 *were found to increase the number of chromosomal breaks by 20%, formation of larger derivative chromosomes by 28%, and reduction in chromatid gaps b by 62% compared to control samples [31]. Thus, chromosomal aberrations are more likely to occur in *MCTS1*-overexpressing cells.

MCTS1 is known to reduce cell sensitivity to etoposide, an inhibitor of topoisomerase II. In order to compare the sensitivity of *MCTS1*-overexpressing cells to the genotoxic effect of etoposide, the DNA comet assay, which allows one to determine the frequencies of DNA double-strand breaks and its repair, was used. Etoposide-treated cells overexpressing *MCTS1 *turned out to have a shorter DNA comet tail, which indicates a more efficient path of repair processes compared to control cells expressing low levels of *MCTS1 *[31]. A decreased* MCTS1 *expression was also shown to activate proteolytic cleavage of poly (ADP-ribose) polymerase (PARP) and reduce its activity. PARP is one of the main proteins responsible for DNA repair, including those associated with the effect of chemotherapeutic drugs [41]. It should be noted that PARP inhibitors are considered promising agents against a number of malignancies [42, 43, 44].

## EFFECT OF MCTS1 ON AKT AND SRC SIGNALING

**Fig. 4:**
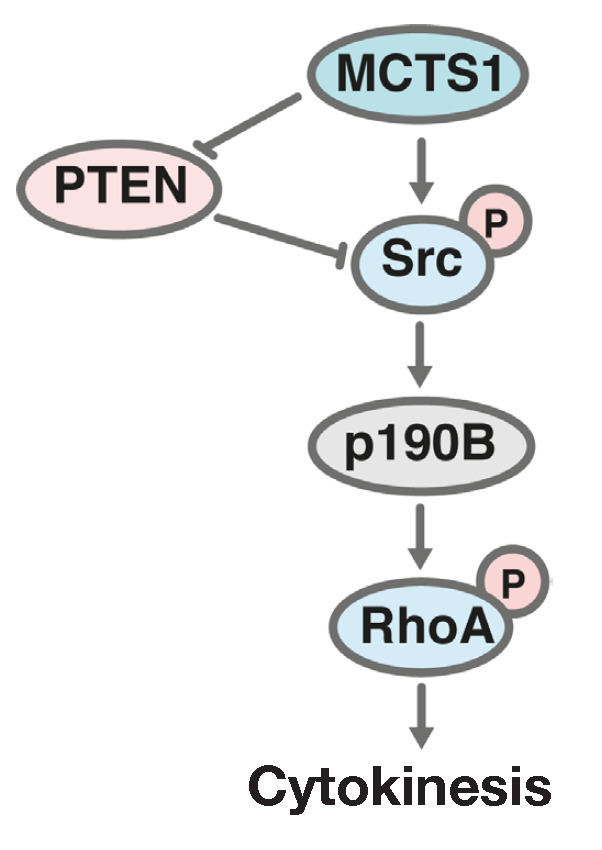
Effect of MCTS1 on the PTEN/Src signaling. PTEN – an inhibitor of the PI3K/AKT/mTOR signaling pathway; Src – a protein kinase of the Src kinase family; RhoA – a transforming protein of the Ras family of GTPases

Protein phosphatase PTEN is one of the main elements in the negative regulation of the AKT signaling pathway (protein kinase B). PTEN damage resulting from mutations or a significant decrease in protein expression can cause malignant cell transformation [45, 46, 47, 48, 49]. Ectopic expression of *MCTS1 *in the human breast cancer cells MCF-10A decreases the levels of PTEN mRNA and protein [40]. An increase in *MCTS1 *expression is accompanied by PTEN degradation. MCTS1 also stimulates the interaction between the Src and p190B proteins, resulting in the formation of a complex inhibiting RhoA, one of the main factors regulating cytokinesis (*Fig. 4*) [50].

MCTS1 is known to regulate not only Src, but the Shc–Ras–ERK signaling pathway as well. Shc (transforming protein 1 with an Src homology domain) is an adaptor protein involved in signal transduction upon activation of certain receptors [51]; in particular, the epidermal growth factor receptor (EGFR) [52], erbB-2 receptor [53], and insulin receptor [54]. Several isoforms of the Shc protein are usually present in cells. An excessive Shc level is associated with abnormal activation of the ERK signaling pathway [55], which, in turn, significantly affects the development and progression of malignancies, including the sensitivity of malignant cells to chemotherapeutic drugs. Suppression of *MCTS1 *expression in immortalized cell lines of breast and lung cancer by RNA interference decreases the levels of p66, p52, and p46 isoforms of the Shc protein [35]. The direct effect of MCTS1 on the signaling pathway involving Shc may partially explain how the increase in *MCTS1* expression associated with the induction of cyclin D1 accumulation and activation of the Rb protein phosphorylation impact on the acceleration of the G1 phase progression (*Fig. 2*).

## MCTS1 ROLE IN THE IL-6/IL-6R SIGNALING PATHWAY

**Fig. 5:**
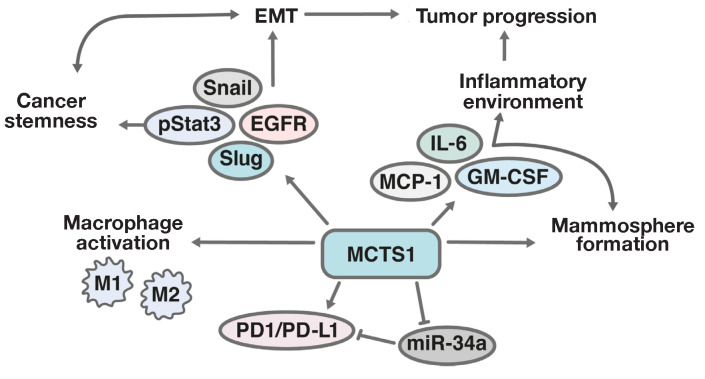
Schematic illustration of the contribution of MCTS1 to EMT, tumor escape from immune surveillance, and activation of pro-inflammatory factors by tumor cells. EMT – epithelial-mesenchymal transition; Snail and Slug – transcription factors involved in EMT; EGFR – epidermal growth factor receptor; pro-inflammatory cytokines IL-6 – interleukin-6, MCP-1 – monocyte chemoattractant protein, and GM–CSF – granulocyte-macrophage colony-stimulating factor; M1 – classically activated macrophages providing the production of pro-inflammatory cytokines; M2 – macrophages responsible for anti-inflammatory response

The IL-6/STAT3 signaling pathway is known to be involved in the regulation of breast cancer cell stemness [56]. Ectopic expression of *MCTS1 *in the human breast cancer cells MDA-MB-231 stimulates the formation of malignant, discrete clusters of cells, namely mammospheres, upon cell growth under certain conditions. It is important that elevation of *MCTS1 *expression increases the level of CD44, a tumor stem cell marker [57]. Treatment of the cells ectopically expressing* MCTS1 *with the IL-6 cytokine leads to an even more rapid formation of mammospheres; therefore, MCTS1 may be involved in the regulation of IL-6 signaling (*Fig. 5*). Treatment of cells with tocilizumab, a monoclonal antibody that inhibits the IL-6 receptor, reduces the intensity of mammosphere formation under* MCTS1 *induction and also significantly decreases the number of cells CD44+/CD24- subpopulation to a control level [57]

A study of the relationship of MCTS1 and IL-6 with the clinical path of the disease revealed a positive correlation between the proteins levels in all patients with triple-negative breast cancer with a deficient expression of the epidermal growth factor receptor (HER2), estrogen receptor (ER), and progesterone receptor (PR). Moreover, high *MCTS1 *and *IL-6 *levels were found to correlate with the risk of metastases [57].

Cytokines and growth factors produced by cells of the tumor microenvironment play an important role in tumor progression [58, 59, 60]. Triple-negative breast cancer cells with enhanced *MCTS1 *expression secrete significantly more of the pro-inflammatory cytokines IL-6, MCP-1, and GM-CSF than cells with a relatively lower *MCTS1 *expression level [57].

## MCTS1 AND IMMUNE SURVEILLANCE OF A TUMOR

When developing approaches to the immunotherapy of malignancies, various methods, such as the receptors and ligands regulating immune surveillance, are used to inhibit immune checkpoints [61]. Currently, one of the most studied mechanisms is based on inhibiting the PD1 receptor and its ligand, PD-L1. An increased PD-L1 level is observed in many oncological diseases. An abnormally high expression of this ligand on the surface of malignant cells is considered to be associated with their evasion of immune surveillance [62, 63]. Anti- PD1/PD-L1 antibodies have been approved for the treatment of certain cancers (melanoma, non-small cell lung cancer, Hodgkin lymphoma, bladder cancer, renal cell carcinoma, squamous cell carcinoma of the head and neck, breast cancer, Merkel cell carcinoma, hepatocellular carcinoma, and stomach cancer) [62]. However, the use of anti-PD1/PD-L1 antibodies turned out to be effective only in some patients and does not always lead to the desired result. MicroRNA miR-34a is involved in the regulation of the PD-L1 signaling pathway [64, 65]. An increase in miR-34a expression in cancer cells causes a pronounced antitumor effect [65].

MCTS1 can induce PD-L1 expression while decreasing miR-34a levels. miR-34a can inhibit the epithelial- mesenchymal transition (EMT) induced upon activation of the TGF-β (transforming growth factor β) signaling pathway [66]. In addition, miR-34a negatively affects the expression of the genes coding for the proteins involved in EMT (Snail, Slug, and ZEB1), as well as the proteins associated with the maintenance of tumor stem cells (BMI1, CD44, CD133, OLFM4, and c-MYC) [67]. In addition, miR-34a is directly involved in the regulation of macrophage activation in the tumor microenvironment and closely related to the immune response to tumor cells. All of this suggests that *MCTS1* suppression, combined with *miR-34a *gene activation, can be considered as a promising strategy in breast cancer therapy.

## ROLE OF MCTS1 AND DENR IN MALIGNANCIES

The hypothesis on the involvement of *MCTS1 *in the malignant transformation of lymphoid cells was suggested almost immediately after the discovery of this gene. Abnormal *MCTS1 *amplification was noted in various malignant lymphoid cell lines. In normal lymphoid tissues, the *MCTS1 *gene is expressed at a low level [67].

An increase in *MCTS1 *expression was found in IL- 2-independent, but not in IL-2-dependent, T-cell lines, including IL-2-stimulated peripheral blood lymphocytes (PBLs) [67]. A high level of *MCTS1 *expression was also observed in a number of transformed B-cell lines derived from patients with non-Hodgkin lymphoma [67].

Thus, an increased level of *MCTS1 *was found in 41% of diffuse large B-cell lymphoma patient samples. However, expression of *MCTS1 *was not observed in chronic lymphocytic leukemia cells [67].

Increased *MCTS1 *expression was later shown to be typical not only of malignant lymphoid diseases.

The study using the Kaplan–Meier method (kmplot.com) demonstrated that high *MCTS1 *expression in breast cancer samples is associated with a lower overall survival rate in patients compared to relatively lower *MCTS1 *levels. This is typical of *TP53*-positive breast cancers, lymph node metastases-free breast cancers, *HER2*-negative breast cancers, luminal-A, and luminal-B breast cancers. Patients with relatively high* MCTS1 *levels in biopsies have lower recurrence-free survival rates compared to patients with a low *MCTS1* expression.

Elevated *MCTS1 *levels were also detected in lung cancer samples. Moreover, high expression levels were noted for all four stages of the disease [57].

Bioinformatic analysis of the transcriptome of tumor cells derived from patients with lung cancer, stomach cancer, hepatocellular carcinoma, and kidney cancer showed that low *DENR *levels correlate with a more favorable disease path and better prognosis [68]. Gene Set Enrichment Analysis (GSEA) showed that *DENR* can be associated with the regulation of the signaling cascades responsible for cell cycle progression, DNA repair, and splicing [68]. Analysis of *DENR *expression in lung cancer metastases showed that a higher gene expression level is characteristic of lymph node metastases.

Detection of the tumor marker alpha-fetoprotein in the blood serum is widely used in the diagnosis of malignancies. An increased level of alpha-fetoprotein is found in blood serum for liver, breast, stomach, and sometimes lung cancer [69, 70]. High serum alphafetoprotein levels are associated with a poor prognosis in patients with hepatocellular carcinoma [71]. A bioinformatic analysis of the transcriptome databases of patients with various oncological diseases revealed that a high level of *DENR *expression in tumor cells correlates with a high serum level of alpha-fetoprotein [68].

Higher *DENR *levels are characteristic of later stages of various tumors, including hepatocellular carcinoma, lung, breast, kidney, and rectal cancer. One study noted that a relatively higher *DENR *expression might indicate an increased risk of glioma in dogs [72]. This was established by a comparative analysis of the transcriptomes of brain samples derived from dog breeds with an elevated risk of developing glioma and breeds less prone to this disease.

The data above indirectly suggest that *DENR *may be associated with the onset and development of oncological diseases and can be directly involved in tumor development. However, it should be noted that most of the data supporting this assumption are obtained using bioinformatics analyses. At the same time, there is nary data to indicate the functional effect of this protein on cellular growth and their sensitivity to chemotherapeutic drugs. It should be also noted that, currently, there is a relatively scarce amount of data describing the involvement of *DENR *in the regulation of the expression of the genes involved in the development of malignant diseases.

These data indicate that the DENR and MCTS1 proteins can be considered promising diagnostic and therapeutic targets.
